# Predictors of alcohol use disorder in patients with hypertension: a national registry-based cohort study

**DOI:** 10.1186/s12889-025-23579-2

**Published:** 2025-07-03

**Authors:** Jørgen G. Bramness, Torgeir Gilje Lid, Ingeborg Bolstad, Lars Lien, Dawit S. Abebe

**Affiliations:** 1 Institute Clinical of Medicine, UiT The Arctic University of Norway, Tromsø, Norway; 2 Section for Clinical Addiction Research, Oslo University Hospital, Oslo, Norway; 3 Norwegian National Advisory Unit on Concurrent Substance Abuse and Mental Health Disorders, Innlandet Hospital Trust, Brumunddal, Norway; 4 Department of Alcohol, Tobacco and Drugs, Norwegian Institute of Public Health, P.O.Box 222, 0213 Skøyen, Oslo, Norway; 5 Centre for Alcohol and Drug Research, Stavanger University Hospital, Stavanger, Norway; 6 Faculty of Health Sciences, University of Stavanger, Stavanger, Norway; 7 Faculty of Social and Health Sciences, University of Inland Norway, Elverum, Norway; 8 Department of Nursing and Health Promotion, Oslo Metropolitan University, Oslo, Norway

**Keywords:** Alcohol use, Alcohol use disorder, Hypertension, Risk, Comorbidity

## Abstract

**Background:**

Alcohol use disorder (AUD) is often discovered very late or not at all, with a risk of undertreatment. High alcohol consumption may lead to hypertension. and hypertensive patients should be asked about their alcohol use. Our aim was to look at additional risk factors for receiving a diagnosis of AUD in patients with hypertension.

**Methods:**

Using the Norwegian full coverage patient registry, we investigated all patients who received an ICD-10 diagnosis of hypertension and who were routinely registered in the specialized healthcare services in 2010. These were followed for six years and those who subsequently received an ICD-10 AUD diagnosis were identified. Age, sex and comorbidities found in the patient registry were used as covariates.

**Results:**

Of the 103 623 patients diagnosed with hypertension in 2010, 1517 (1.46%) were later diagnosed with AUD. Male sex, young age, having several comorbidities, malnutrition and respiratory disease, anxiety, and especially a diagnosis of major depression increased the risk of being diagnosed with AUD in these hypertensive patients.

**Conclusion:**

Only a small proportion of the hypertensive patients were later diagnosed with AUD, reflecting that patient registries are poor indicators of true AUD prevalence, since AUD is heavily underdiagnosed. However, the current findings suggest that comorbid conditions such as depression, anxiety, malnutrition, and respiratory disease in hypertensive patients should prompt clinicians to assess alcohol use more proactively. This could in turn lead to better care for both those with hypertension and alcohol problems.

**Supplementary Information:**

The online version contains supplementary material available at 10.1186/s12889-025-23579-2.

## Introduction

Risky alcohol use and alcohol use disorder (AUD) are leading causes of morbidity and mortality worldwide [1]. Nevertheless, problematic alcohol use and AUD often go unrecognized, undiagnosed, and untreated [2]. withTime from onset of AUD to treatment maybe as much as two decades [3], delayed by inaction on the part of both clinicians and patients. Many doctors hesitate to ask patients about their alcohol use since it is perceived to be a personal and sensitive issue, thereby missing opportunities to intervene in a major risk factor of illness [4]. Patient’s inaction may be related to an unawareness of the problem and of possible available treatments. There may also be financial barriers, low trust in the help being offered, stigma related to AUD, or a belief that the problem will resolve without specific treatment [5]. Since alcohol use is often not addressed, patients do not receive relevant advice on how their alcohol consumption may influence their health [6]. Earlier acknowledgement and intervention for risky alcohol use or AUD could benefit patients, both in terms of adequate treatment for AUD and for their general health.

Hypertension is a common condition and a leading cause of morbidity and mortality [7]. Alcohol use, with or without AUD, is a risk factor for hypertension, but the relationship between alcohol use and hypertension is complex [8]. Blood pressure will fall swiftly after intake of alcohol, and thus consuming low levels of alcohol may have a beneficial effect on blood pressure [9]. This differs with the long-term effects however [10], where high alcohol use is a risk factor for hypertension [11]; with every unit of daily alcohol intake, blood pressures increases by 1.25 mm Hg [12]. We also know that hypertension is common among patients with AUD [13]. The population attributable fraction for hypertension contributed by heavy drinking is 3–12% [14], thereby indicating that alcohol use makes a moderate contribution in causes of hypertension. Still, there is a well-documented causal effect of chronic high alcohol consumption upon hypertension, and indications of increased mortality from hypertension among binge drinkers [15, 16]. Despite this there are few, if any, studies documenting the prevalence of AUD among patients with hypertension, with one of few exceptions being a study from Cape Verde finding AUD among approximately 14% of the alcohol consumers with hypertension [17].

Even though both AUD and hypertension are frequent health problems in Western societies, and the relationship between them is well established, the low awareness of this in clinical settings results in delays to diagnosis and suboptimal treatment of both [8]. Population-based studies have shown that a diagnosis of hypertension does not lead to significant changes in drinking behaviour, probably caused by little attention to alcohol in most hypertension management guidelines. This is true even where physicians in some countries have received recommendations to enquire about alcohol when encountering hypertension [8, 18]. For example, in Sweden, the blood test for phosphatidyl ethanol (PEth), a highly significant and specific marker of alcohol use, is increasingly used as a routine test in primary care for patients with hypertension [19].

In general, patients will often come to their doctor’s office with conditions or diseases associated with the use of alcohol rather than the alcohol problem itself, and the potential relation to alcohol consumption may remain unnoticed both by the patient and the doctor. One approach to improve detection of risky alcohol use and AUD is to systematically address alcohol use where such symptoms or diseases appear. Hypertension is just one of the adverse effects found in patients with high use of alcohol. Others may include metabolic disorders, coronary heart disease, respiratory diseases cardiomyopathies, low back pain, anxiety disorder, depression, insomnia, frequent absences from work due to sickness or failing to maintain employment [1, 20].

Pragmatic approaches to addressing alcohol use in clinically relevant situations have been suggested and to some extent found to be superior to universal screening strategies in identifying patients with alcohol-related health problems or AUD [21–23]. Training and support that focus on brief interventions and health problems related to alcohol may be cost-effective and improve practice in primary care [24]. Implementation of such strategies for alcohol screening and brief interventions have been mostly studied in primary care, and the individual and system-related barriers are numerous and well documented [25]. However, the relationship between alcohol use with or without AUD and hypertension may form a basis for a clinically relevant and feasible practice improvement, transferable to other clinical settings. Routinely addressing alcohol for all patients with hypertension, and thereafter repeating and expanding the assessment for treatment resistant hypertension, with or without the use of PEth, is probably a feasible and acceptable strategy [8, 19].

The present study aimed to identify sociodemographic and comorbid clinical predictors of a later diagnosis of AUD in patients with diagnosed hypertension in specialized health care using nationwide patient registry data.

## Methods and materials

This is a register-based cohort study combining sociodemographic information from Statistics Norway and information on physical diseases and mental disorders obtained from the Norwegian Patient Registry (NPR). NPR holds data on all registered diagnoses obtained during contacts with specialist health care services. The unique national identity number assigned to each person enables tracking of the same patient over time from hospital to hospital and analysing their data without concern of duplicates of hypertension diagnosis. All diagnoses are based on the International Classification of Diseases 10th Revision (ICD-10) codes [26].

We identified the study population consisting of all adults 18 years or older who were legal residents in Norway starting from January 1st, 2008, to December 31st, 2016 (*N* = 4 652 365). Patients with a diagnosis of hypertension (ICD-10 I10-I15) (*N* = 168 322) as primary or secondary diagnosis were identified during the year of 2010. We assigned a washout period of 2 years (2008–2009) prior to the diagnosis for eliminating those who already had received a diagnosis of alcohol use disorder (AUD; ICD-10 F10; *N* = 2046). Patients who were registered as deceased (*N* = 63,414) during the study period (2008–2016) were excluded from analysis. The final sample included for analysis consisted of 103 623 patients diagnosed with hypertension that were followed until a diagnosis of AUD during the study period– January 1, 2010, through December 31, 2016. The data were dichotomized to hypertension patients who did receive a diagnosis of AUD in the follow-up period vs. those who did not.

### Explanatory variables: mental and somatic disorders

Supplementary Table S1 presents the ICD-10 codes of a range of mental disorders, cardiovascular diseases, metabolic disorders, and respiratory diseases. For mental disorders, the codes include F32-F34 for major depression, F60-F69 for personality disorders, F43 for adjustment disorders, F40-F41 for anxiety disorders, and F30-F31 for bipolar disorder. Cardiovascular diseases are categorized with codes I20-I25 for ischemic heart disease and I61-I67 for cerebrovascular diseases. Metabolic disorders were identified with the codes E70-E90, with E10-E11 for diabetes mellitus, E66 for obesity and E46 for malnutrition. Respiratory diseases include J40-J44 for chronic lower respiratory diseases, J00-J06 for acute upper respiratory disorders, and J11 for influenza and pneumonia. These classifications provided a structured approach for analysing the comorbid conditions present in hypertensive patients. These variables were selected based on prior research evidence linking them to both AUD and hypertension [1, 20]. This literature-informed approach allowed us to prioritize clinically meaningful covariates while ensuring consistency with prior studies.

#### Covariates

age and sex were used as covariate variables. The age variable (per 1. January 2008) was used as a continuous variable. Sex was coded 0 for males and 1 for females.

### Statistical analyses

The study utilized Cox proportional regression models to estimate the risks of diagnosed AUD among patients with hypertension. Hazard ratios (HRs) with 99% confidence intervals (CIs) were reported, and statistical significance was determined by *p*-values ≤ 0.01. Estimates were adjusted for age and sex. The proportional hazard assumptions were tested using Schoenfeld residuals after fitting each model (“*estat phtest tests”*) and the covariates were found to meet the proportional hazards assumption. The final models were decided using Akaike and Bayesian information criterion (AIC and BIC), where a lower value indicates a better model fit. All analyses were performed using Stata SE/18 (StataCorp., USA, 2023).

## Results

Table 1 presents the demographics and comorbidities of hypertensive patients who were later diagnosed with AUD compared to those who were not. Patients were followed from the date of hypertension diagnosis in 2010 until the first occurrence of an AUD diagnosis or the end of the study period on December 31, 2016. Individuals who did not receive an AUD diagnosis were censored at the end of follow-up. Of the 103 623 patients diagnosed with hypertension in specialized health services in 2010, 1517 (1.46%) were diagnosed with an AUD in the follow up period 2011–2016. Being female was associated with a significantly lower risk of receiving an AUD diagnosis, with only 26% of females in the AUD group compared to 50% in the non-AUD group. Among those with hypertension, 0.6% of the females and 2.2% of the males later received a diagnosis of AUD (data not shown in table). Age also played a role, with a mean age of 59 years in the AUD group versus 63 years in the non-AUD group. The adjusted estimates showed that all mental and somatic diseases significantly increased the risk of AUD, except for acute upper respiratory disorder. Specifically, mental disorders such as major depression (Risk ratio (RR) = 5.25), personality disorders (RR = 4.84), and bipolar disorder (RR = 5.37) significantly increased the risk of AUD. For somatic disorders, malnutrition showed the highest relative risk (RR = 3.70), followed by chronic lower respiratory disease (RR = 2.03), indicating that these comorbidities are strong predictors of being diagnosed with AUD in hypertension patients. This universal increase in risk was reflected in an increased risk of AUD of almost 40% for every new comorbid diagnosis added.

**Table 1:** The number and percentage (N (%)) of the patients diagnosed with hypertension that later are diagnosed with alcohol use disorder (AUD), according to sex, age, and comorbid disorders. All risk ratios (RR) are calculated and presented as unadjusted figures and adjusted for age and sex

	Hypertensive patients				
	Later AUD	No later AUD			
	*N* = 1 517	*N* = 102 106		RR (95% CI)	
	1.46%	98.54%		unadjusted	Adjusted for age and sex
Sociodemographic					
Sex (female)	349 (25.97)	51 056 (50.00)		0.35 (0.31–0.39) ***	0.40 (0.35–0.45) ***
Age (years) ^a^	59 (11)	63 (12)		0.96 (0.95–0.96) ***	0.96 (0.96–0.97) ***
Mental disorders					
Major depression	386 (6.14)	1131 (1.16)		5.28 (4.72–5.91) ***	5.25 (4.72–5.88) ***
Personality disorders	76 (10.09)	1441 (1.40)		7.20 (5.78–8.97) ***	4.84 (3.88–6.03) ***
Adjustment disorders	138 (6.37)	1379 (1.36)		4.68 (3.95–5.55) ***	3.91 (3.29–4.63) ***
Anxiety disorders	334 (5.20)	1183 (1.22)		4.27 (3.79–4.81) ***	4.09 (3.62–4.62) ***
Bipolar disorder	70 (8.89)	1447 (1.41)		6.32 (5.02–7.95) ***	5.37 (4.29–6.72) ***
Cardio-vascular disease					
Ischemic heart disease	537 (1.48)	980 (1.46)		1.01 (0.91–1.12)	1.09 (0.98–1.22)
Cerebrovascular diseases	323 (1.92)	1194 (1.37)		1.39 (1.23–1.58) ***	1.69 (1.49–1.91) ***
Other forms of heart disease	630 (1.49)	887 (1.45)		1.02 (0.92–1.13) ***	1.28 (1.15–1.42) ***
Metabolic					
Metabolic disorder	726 (2.35)	791 (1.09)		2.15 (1.95–2.37) ***	1.15 (1.95–2.37) ***
Diabetes mellitus	415 (1.71)	1102 (1.39)		1.23 (1.10–1.37) ***	1.16 (1.03–1.29) **
Obesity	187 (2.31)	1330 (1.39)		1.66 (1.42–1.93) ***	1.26 (1.08–1.47) ***
Malnutrition	100 (3.95)	1417 (1.40)		2.81 (2.30–3.43) ***	3.70 (3.04–4.51) ***
Respiratory disease					
Chronic lower respiratory disease	374 (2.29)	1143 (1.31)		1.74 (1.55–1.96) ***	2.03 (1.80–2.27) ***
Acute upper respiratory disorder	43 (1.69)	1474 (1.46)		1.15 (0.83–1.57)	1.13 (0.83–1.52)
Influenza and pneumonia	296 (2.16)	1221 (1.36)		1.58 (1.40–1.80) ***	1.96 (1.73–2.23) ***
Number of diagnoses	3.05 (1.93)	2.01 (1.61)		1.36 (1.32–1.39) ***	1.38 (1.35–1.41) ***

***p-value < 0.001; **< *p*-value < 0.01, ^a^ for this variable the figures indicate mean and standard deviation

Supplementary Table S2 evaluates the model fit for predicting AUD in patients diagnosed with hypertension by comparing various models with AIC and BIC values. The null model had the highest AIC and BIC values, serving as a baseline. The AIC and BIC results indicate that including comorbid mental and somatic disorders significantly enhances the accuracy of identifying patients at risk for AUD. The most substantial improvement was observed when major depression was added, reducing the AIC by 1,247 points and the BIC by 1,218 points. Anxiety disorders and metabolic disorders also significantly improved the model fit. These results indicate that including comorbid mental health conditions in the predictive models greatly enhances the accuracy of identifying patients at risk for AUD.

**Fig. 1:**
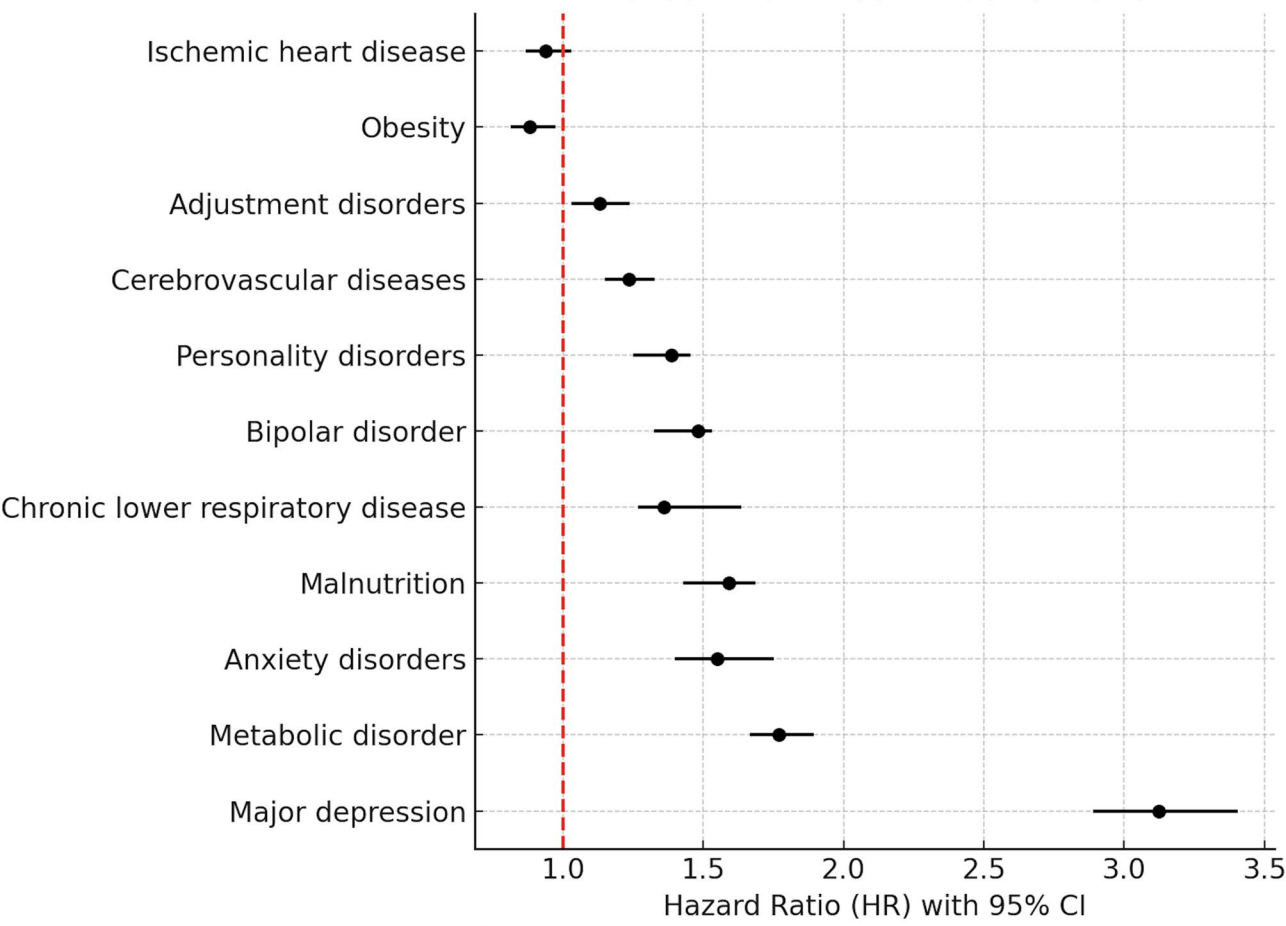
The forest plot summarizing HR with 95% confidence intervals for each predictor of alcohol use disorder in patients with hypertension

Table 2 provides the results of a stepwise regression analysis, identifying variables that significantly influence the risk of AUD in hypertension patients. All mental and somatic disorders listed in Table 2, except for obesity and ischemic heart disease, significantly predict the risk of AUD in hypertensive patients. Specifically, major depression emerged as the most influential factor, increasing the risk of AUD more than threefold (HR = 3.1, z-score = 27.1). Metabolic disorders (HR = 1.8, z-score = 17.2), anxiety disorders (HR = 1.6, z-score = 9.2), and malnutrition (HR = 1.6, z-score = 9.1) also significantly raised the risk, accordingly. Interestingly, comorbid obesity was associated with a decreased risk of being diagnosed with AUD (HR = 0.883, z-score = -1.9). A summary of these results is presented in the forest plot (Fig. 1). Results (HR with 95% CI) for sex-specific predictors of AUD in patients with hypertension are presented in Supplementary Table S3.

**Table 2:** Stepwise regression estimates showing variables that are considered more influential in predicting alcohol use disorder in patients with hypertension

	HR	95% CI		*p*-value	z
Major depression	3.123	2.888	3.406	< 0.001	27.07
Metabolic disorder	1.769	1.664	1.894	< 0.001	17.23
Anxiety disorders	1.549	1.397	1.751	< 0.001	9.21
Malnutrition	1.591	1.429	1.685	< 0.001	9.08
Chronic lower respiratory disease	1.360	1.269	1.637	< 0.001	8.85
Bipolar disorder	1.480	1.324	1.532	< 0.001	7.27
Personality disorders	1.385	1.251	1.456	< 0.001	6.34
Cerebrovascular diseases	1.236	1.150	1.326	< 0.001	5.85
Adjustment disorders	1.130	1.030	1.239	0.013	2.47
Obesity	0.883	0.814	0.973	0.007	-2.75
Ischemic heart disease	0.937	0.867	1.031	0.051	-1.95

Backward selection was applied. Estimates were age and sex adjusted. Variables with *p*-value > 0.2 were excluded (i.e., diabetes & influenza). No multicollinearity problem (i.e., VIF < 5 for all variables except age and sex). HR = Hazard ratio; CI = Confidence Interval

## Discussion

In this observational register-based investigation of patients in specialized health care diagnosed with hypertension, male sex and young age further increased the risk of receiving a subsequent diagnosis of alcohol use disorder (AUD). An increased risk for AUD was also found among the hypertension patients with comorbid disorders like mental disorders, malnutrition, respiratory disease and among those with multiple diagnoses. Models looking at all these risk factors together revealed that having a diagnosis of major depression, metabolic disorder, anxiety disorder and malnutrition increased the risk of receiving a diagnosis of AUD with each additional disorder added.

The underlying intention of the current study was that by being attentive towards patients with hypertension, we could establish a means of early detection and treatment of risky alcohol use and AUD, at the same time as improving the treatment of hypertension. Routine alcohol screening in primary care settings is underutilized, as confirmed by population studies and studies exploring health care professionals’ experiences [27, 28]. A more pragmatic approach based on disorders where alcohol use is clinically relevant could lead to more patients with risky alcohol use or AUD being identified and offered help. This will likely be more acceptable to clinicians and patients [21, 22]. Such targeted, evidence-based approaches bear the potential of improving clinical practice, both regarding diagnosis and treatment of AUD, and regarding treatment and follow-up of the clinical conditions related to alcohol use.

Looking for situations where such a pragmatic approach could be utilized, hypertension may be an ideal test case, since the relationship between hypertension and alcohol use is well documented. Furthermore, both hypertension and AUD have early stages with varying severity. Prehypertension is not hypertension, and risky drinking is not AUD, but both phenomena may proceed to clinical thresholds, and both may benefit from early identification. Still the population that consumes attributable fraction of alcohol use on hypertension is only moderate [14] and most hypertensive patients do not have an unhealthy level of alcohol use. Even if alcohol use is a major risk factor for AUD, less than 2% of the patients later received a diagnosis of AUD in the present study, more so in males than in females. This is substantially lower than in a previous study, but their methodology allowed for higher detection rates [17]. The low prevalence in the current study prompted us to search for additional indicators to alert the clinician. The current study indicates that young age and male sex in the hypertensive patient should prompt them to enquire about alcohol use. Furthermore, investigating additional comorbid disorders increasing the risk for AUD for patients with hypertension is important to further develop clinical indicators and thus improve practice.

The current study demonstrates that even with a risk factor such as hypertension, very few are diagnosed with AUD. Males were diagnosed with a later AUD 3–4 times more often than females. This is a larger difference than usually quoted in prevalence rates of gender differences in AUD [29]. The low detection and treatment rate is in concordance with former studies [2]. However, the study revealed that hypertensive patients also diagnosed with major depression had a further increased risk of a later AUD diagnosis. Among individuals with major depression, the estimates of comorbid AUD varies from 27 to 40% for lifetime prevalence [30, 31] and up to 22% for 12-month prevalence [32]. A systematic review from 2020 concluded that this association could not be fully explained by common factors affecting both disorders, indicating a causal relationship pathway from alcohol use or AUD to major depression [33]. Even if the current investigation looks at comorbidities that precede AUD it is not possible to draw conclusions about causality, due to the large lag in AUD diagnosis and a circular relationship between these disorders [34]. It is essential to be attentive to signs of mood disorders in those with AUD and vice versa. Converging evidence suggests genetic links between AUD and mood disorder [35, 36].

We also found that comorbid anxiety disorder increased the risk of AUD among the hypertension patients. Generalized anxiety disorder, social anxiety disorder, and panic disorder often co-occur with AUD [37], with prevalence of AUD among individuals treated for anxiety disorders ranging from 20 to 40% [32, 38]. This rate is significantly higher than that observed in the general population, suggesting that anxiety disorders are a risk factor for AUD, but can also follow AUD. Genetic and environmental factors also contribute to the concurrent occurrence of AUD and anxiety disorders [39]. Since alcohol is readily available, it is frequently used to cope with anxiety. While alcohol may appear to alleviate anxiety in the short term, prolonged heavy drinking and repeated abstinence can exacerbate both anxiety symptoms and maladaptive drinking behavior [37].

In the present study both malnutrition and metabolic syndrome were linked to a later AUD diagnosis in the hypertensive patients. It is well known that heavy alcohol use and AUD is linked to malnutrition, especially in the later stages of AUD, since heavy alcohol use may disturb healthy eating habits and lead to lower intake of essential nutrients [40]. Metabolic syndrome was also related to a later diagnosis of AUD in this hypertension group of patients, a finding that coheres with observations elsewhere [41]. Hypertension itself may be a part of metabolic syndrome. In the current study and in bivariate analysis we found an increased risk of AUD for obesity, repeating findings from previous studies [42]. However, in the multivariate analysis, being diagnosed with obesity *decreased* the risk of being diagnosed with AUD, probably due to the interaction with other predictors.

We also found that pulmonary disorder was associated with a later diagnosis of AUD in hypertension patients. This is in line with findings that indicate that there is an increased risk of AUD in people with chronic obstructive pulmonary disease [43], and other pulmonary diseases [20].

Lastly, we found that receiving multiple diagnoses increased the risk of later being diagnosed with AUD. The fact that alcohol use and AUD is associated with more than 200 diseases or conditions [1] and that many AUD patients go many years undetected might explain this finding. From studies investigating comorbidities of AUD, we know that more than half have multiple somatic disorders [44].

### Limitations

This observational study draws its information from the Norwegian national health registry covering the entire population, but with unknown transferability to other nations. Further, it covers only specialized health care. This means that people who are not diagnosed with hypertension or AUD or any of the comorbidities in specialized health service are not recorded. We know that there is a low treatment coverage of AUD in specialized health care [2] and that most patients with hypertension are treated in primary health care [45]. It is reasonable to assume that only the most severe cases are treated in specialized health care, but also that the undertreatment is larger in AUD than in hypertension, leading to an underestimation of the observed rates, a point that is supported by other studies finding higher rates of AUD in hypertension patients [17]. Also, we have no information about elevated blood pressure that does not reach diagnostic level and the same with risky alcohol use, also probably leading to an underestimation of the relationship between the two. The intention of the current paper is, however, not to identify the prevalence of AUD or hypertension, but rather to point to risk factors among hypertensive patients to be considered when predicting AUD. Furthermore, the diagnoses are clinical ones, and we do not know if guidelines have been followed in all cases, thus leaving questions on the accuracy of the diagnoses. Also, we have quite “shallow data”, with no information on the actual blood pressure, the cause of the increased blood pressure, nor the level of alcohol use. All this leaves us to suggest that the observed relationships are only indications of what goes on in real-life situations. Lastly, we included only living patients, whilst deceased during the observation period were omitted since we had no information on cause of death in the current material. It is, however, a possible speculation that those who are later diagnosed with AUD have a higher mortality and this might have led to an underestimation of the effects.

## Conclusion

In this study, we found that in patients diagnosed with hypertension in specialized health care, having a comorbid diagnosis of a mental disorder like major depression or anxiety, or a somatic disorder like malnutrition or metabolic disorder, substantially increased the risk of later receiving a diagnosis of AUD. This was especially true for younger males. Such combinations of diagnoses should increase the medical doctor’s awareness of the need to ask about alcohol use and alcohol use problems in patients with hypertension, allowing for an advantageous earlier detection of problematic alcohol use or AUD. The figures in the present study are only on diagnosed hypertension and AUD and so represent only the most severe cases, thus gravely underestimating the effect sizes. Our findings were from Norwegian specialized health care, and we do not know the true validity for primary health care, nor those outside Norway. Nevertheless, it is reasonable to assume that the results are valid also in other settings and as such supports a possible integration of structured assessment of alcohol use into the routine care of hypertensive patients, particularly when multiple or specific comorbidities and demographic risk profiles are present.

Given that most cases of both hypertension and AUD are found and treated in primary health care, future research in this area should incorporate data from this setting. Also, the use of registry data from more countries and including socioeconomic status as a covariate could enhance the generalizability of findings across diverse healthcare systems, making the finding valid also outside of Norway. The use of more primary data, e.g., alcohol use parameters like self-reported alcohol use or PEth and measured blood pressure could reduce misclassification bias. Other designs like Mendelian randomization or quasi-experimental designs could further enhance the research that could also include data on medication adherence and polypharmacy in the AUD-hypertension comorbidity.

## Electronic Supplementary Material

Below is the link to the electronic supplementary material.

Supplementary Material 1

## Data Availability

The data that support the findings of this study are available from the Norwegian Patient Registry (NPR) but restrictions apply to the availability of these data, which were used under license for the current study, and so are not publicly available. Data are however available from the authors upon reasonable request and with permission of NPR.

## Abbreviations

AIC: Akaike information criterion
AUD: Alcohol use disorder
BIC: Bayesian information criterion
CI: Confidence interval
HR: Hazard ratio
ICD-10: International classification of diseases 10th revision
PEth: Phosphatidyl ethanol
RR: Relative risk
