# Supplementary material for: Predictors of alcohol use disorder in patients with hypertension: a national registry-based cohort study
Source: BMC Public Health. 2025 Jul 3;25:2352. doi: 10.1186/s12889-025-23579-2 (PMC12224397; doi:10.1186/s12889-025-23579-2)
Supplement: Supplementary file 1 — Supplementary Material 1 [file 12889_2025_23579_MOESM1_ESM.docx]

**Supplementary table S1.** ICD-10 ^a^ codes for explanatory variables (mental disorders and somatic diseases)

| Variables | ICD-10 codes |
| --- | --- |
| Mental disorders |  |
| Major depression | F32-F34 |
| Personality disorders | F60-F69 |
| Adjustment disorders | F43 |
| Anxiety disorders | F40-F41 |
| Bipolar disorder | F30-F31 |
| Cardio-vascular disease |  |
| Ischemic heart disease | I20-I25 |
| Cerebrovascular diseases | I61-I67 |
| Other forms of heart disease | I30-I52 |
| Metabolic |  |
| Metabolic disorders | E70-E90 |
| Diabetes mellitus | E10-E11 |
| Obesity | E66 |
| Malnutrition | E40-E46 |
| Respiratory disease |  |
| Chronic lower respiratory disease | J40-J4A |
| Acute upper respiratory disorder | J00-J06 |
| Influenza and pneumonia | J09-J18 |

^a^ ICD-10: The International Classification of Diseases, 10th Revision

**Supplementary table S2.** Akaike information criterion (AIC) and Bayesian information criterium (BIC) values showing the best-fit of models in explaining the risk of alcohol use disorder among patients diagnosed with hypertensive disorder.

| Models | AIC | Difference from the null model | BIC | Difference from the null model |
| --- | --- | --- | --- | --- |
| Null model | 15829 | - | 15838 | - |
| Age and sex | 15174 | -655 | 15202 | -636 |
| Major depression | 14582 | -1247 | 14620 | -1218 |
| Anxiety disorders | 14787 | -1042 | 14825 | -1013 |
| Adjustment disorders | 15008 | -821 | 15046 | -792 |
| Bipolar disorder | 15050 | -779 | 15089 | -749 |
| Personality disorders | 15054 | -775 | 15093 | -745 |
| Ischemic heart disease | 15713 | -116 | 15211 | -627 |
| Cerebrovascular diseases | 15114 | -715 | 15193 | -645 |
| Other forms of heart disease | 15154 | -675 | 15193 | -645 |
| Metabolic disorder | 14889 | -940 | 14928 | -910 |
| Diabetes mellitus | 15167 | -662 | 15206 | -632 |
| Obesity | 15167 | -662 | 15099 | -739 |
| Malnutrition | 15061 | -768 | 15099 | -739 |
| Chronic lower respiratory disease | 15050 | -779 | 15088 | -750 |
| Acute upper respiratory disorder | 15175 | -654 | 15213 | -625 |
| Influenza and pneumonia | 15081 | -748 | 15119 | -719 |

Estimates for psychiatric and somatic diseases are derived from age and sex adjusted models.

**Supplementary table S3.** Stratified regression estimates showing sex-specific predictors of alcohol use disorder in patients with hypertension.

| **Variables** | **Men** | | | **Women** | | |
| --- | --- | --- | --- | --- | --- | --- |
|  | **HR** | **95% CI** | | **HR** | **95% CI** | |
| Major depression | 3.144*** | 2.820 | 3.505 | 3.389*** | 2.916 | 3.938 |
| Metabolic disorder | 1.770*** | 1.625 | 1.928 | 2.092*** | 1.853 | 2.362 |
| Malnutrition | 1.599*** | 1.394 | 1.833 | 1.160 | 0.953 | 1.411 |
| Anxiety disorders | 1.537*** | 1.353 | 1.748 | 2.031*** | 1.736 | 2.376 |
| Bipolar disorders | 1.409*** | 1.213 | 1.636 | 1.872*** | 1.612 | 2.174 |
| Chronic lower respiratory disease | 1.396*** | 1.277 | 1.527 | 1.123 | 0.991 | 1.273 |
| Personality disorders | 1.344*** | 1.168 | 1.546 | 1.202* | 1.023 | 1.413 |
| Cerebrovascular diseases | 1.222*** | 1.114 | 1.341 | 1.572*** | 1.375 | 1.797 |
| Adjustment disorders | 1.150* | 1.011 | 1.308 | 0.983 | 0.858 | 1.127 |
| Ischaemic heart disease | 1.074 | 0.985 | 1.172 | 0.953 | 0.844 | 1.076 |
| Obesity | 0.915 | 0.812 | 1.031 | 0.636*** | 0.530 | 0.764 |
| Acute upper respiratory infections | 0.838 | 0.672 | 1.044 | 1.482*** | 1.209 | 1.815 |

Estimates were age adjusted. HR= Hazard ratio; CI = Confidence Interval; *** p-values <0.001; ** p-value <0.01; * p-value<0.05
